# Real-Time Monitoring and Step-by-Step Guidance for Transapical Mitral Valve Edge-to-Edge Repair Using Transesophageal Echocardiography

**DOI:** 10.1155/2021/6659261

**Published:** 2021-04-21

**Authors:** Zhenyi Ge, Cuizhen Pan, Wei Li, Daxin Zhou, Wenzhi Pan, Lai Wei, Haiyan Chen, Xianhong Shu, Junbo Ge

**Affiliations:** ^1^Department of Echocardiography, Shanghai Institute of Medical Imaging, Shanghai Institute of Cardiovascular Disease, Zhongshan Hospital, Fudan University, Shanghai, China; ^2^Department of Cardiology, Shanghai Institute of Cardiovascular Disease, Zhongshan Hospital, Fudan University, Shanghai, China; ^3^Department of Cardiac Surgery, Shanghai Institute of Cardiovascular Disease, Zhongshan Hospital, Fudan University, Shanghai, China

## Abstract

MitraClip edge-to-edge (E2E) repair system is the only transcatheter device recommended in the current guidelines for treating mitral regurgitation (MR). The percutaneous femoral venous transseptal access of MitraClip requires a complex steerable delivery system and may thus be technically complex to optimally position and deploy the clip onto the mitral valve. A transapical approach for E2E repair has been devised to treat MR for the ease of operation (ValveClamp system, Hanyu Medical Technology, Shanghai). The first-in-human study of ValveClamp has demonstrated its early feasibility and effectiveness for the treatment of patients with degenerative MR. Transesophageal echocardiography (TEE) is the only imaging modality required for intraoperative guidance of ValveClamp implantation. Successful implantation depends on accurate localization and orientation of the clamp and efficient intraoperative communication between the echocardiographer and the intervention team. Thus, the focus of this review is on elaborating how two-dimensional (2D) and three-dimensional (3D) TEE are used in clinical practice to guide ValveClamp implantation and it may facilitate the understanding of simplicity and safety of this novel procedure. We also describe the implementation of several novel advancements in 3D TEE imaging, which improve the confidence of image interpretation for intraoperative guidance and expedite implantation times.

## 1. Introduction

Transcatheter mitral valve (MV) interventions are progressively being introduced into clinical practice targeting a population of patients with mitral regurgitation (MR) deemed inoperable or at a prohibitive surgical risk [1]. Echocardiography is the imaging modality of choice for patient selection, intraoperative guidance, and assessment of procedural effect as well as potential complications for these therapies [2–7].

The MitraClip edge-to-edge (E2E) repair system is the only transcatheter device recommended in the current guidelines for treating MR. [8] The percutaneous femoral venous transseptal access of MitraClip requires a complex steerable delivery system and may thus be technically challenging to optimally position and deploy the clip onto the mitral valve [9].

Recently, a transapical approach for E2E repair has been introduced to treat MR for the ease of operation (ValveClamp system, Hanyu Medical Technology, Shanghai). The first-in-human study of ValveClamp has demonstrated its early feasibility and effectiveness for the treatment of patients with degenerative MR (dMR) [10] and potentially could be used to correct functional MR [11].

Transesophageal echocardiography (TEE) is the only imaging modality required for intraoperative guidance of ValveClamp implantation. Successful implantation depends on accurate localization and orientation of the clamp and efficient intraoperative communication between the echocardiographer and the intervention team.

Transapical E2E repair using ValveClamp has its own distinct characteristics on echocardiographic imaging for intraoperative guidance. While assessment of mechanism and grading of MR, as well as the procedural effect after transcatheter E2E repair, has been well established [12–14], this review aims to elaborate how 2D and 3D TEE are used in clinical practice to guide ValveClamp implantation and discusses their contribution to the simplicity and safety of this novel procedure.

## 2. Transapical Mitral Valve Edge-to-Edge Repair

**Figure 1:**
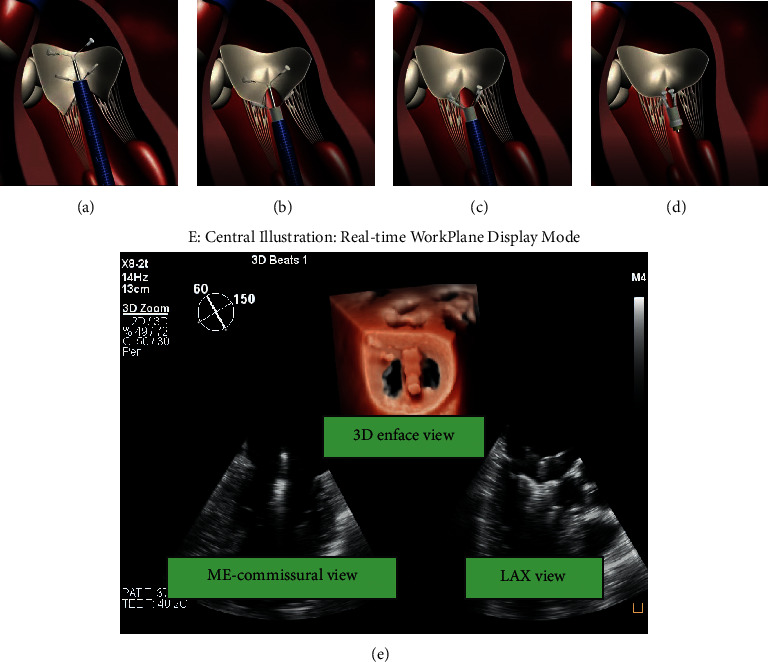
The main steps of ValveClamp implantation. (a) A clamp is delivered to the left atrium. (b) The clamp is adjusted to the appropriate position, and the rear clamp is placed just under the leaflets, while the front clamp remains in the left atrium. (c) The front clamp is pulled back to capture the leaflets, and then the closed ring is moved forward to cover the ventricular end of the clamp arms, making them close to each other. (d) The clamp is released. Reproduced with permission from Pan et al. [10]. (e) Central illustration. The real-time workplane display mode simultaneously depicted X-plane views and 3D enface MV views, which is essential for navigating the main steps of ValveClamp implantation. ME, midesophageal; LAX, long-axis view.

Transapical E2E repair using ValveClamp system involves the introduction of a user-friendly leaflet clamp into the left atrium (LA) and coaxial deployment of the clamp to the mitral valve (Figure 1), which can be easily achieved by selecting an appropriate apex puncture site using 2D and 3D TEE [10].

Transseptal puncture, as well as fluoroscopy guidance, is omitted during the procedure. For percutaneous femoral venous transseptal access repair devices, such as MitraClip and PASCAL, fluoroscopy may provide additional helpful information in regard to the opening, orientation, and adjustment of the device. During ValveClamp implantation, the accurate localization and orientation of the clamp thus completely rely on the intraoperative real-time 2D and 3D TEE imaging.

The key to a successful procedure using ValveClamp is explicit mutual communication between the echocardiographer and interventionist. A real-time display is needed for both sides to view the echocardiographic images.

Recent advancement in TEE (X8-2t transducer, Philips Healthcare, Eindhoven, NL) provides increased resolution and tissue filling in 2D and real-time 3D visualization and meanwhile enables one-beat acquisitions with high volume rates in real-time 3D and 3D color flow Doppler (CFD) imaging. Photo-realistic 3D rendering techniques (TrueVue, Philips Healthcare, Eindhoven, NL) are designed to improve visualization of anatomical structures that appear pink-to-red depending on the intracardiac position of a virtual light source [15, 16].

These novel imaging techniques are now routinely used at our center for guidance in transapical E2E repair procedures and this protocol described in this review article is primarily based on the implementation of these techniques.

## 3. Echocardiographic Protocol for Transapical Mitral Valve Edge-to-Edge Repair

**Step 1 .**

Localization of the cardiac apex

The ValveClamp implantation procedure is performed in the hybrid operating room with patients under general anesthesia. TTE apical 4-chamber view is used to locate the cardiac apex. To obtain this view, the transducer is usually positioned at the left fifth or sixth intercostal anterolateral space. Ideally, it encompasses a full view of all four chambers, including full visualization of the LV to avoid foreshortening. A marker is then made at the site of the probe. After confirmation of the location, a left anterolateral minithoracotomy is performed to expose the apex. In situations that the apex lay under the sixth costa, incision can be made at the fifth intercostal space and the surgeons would use infilling outside the diaphragmatic surface of the heart to facilitate exposure.

**Step 2 .**

Transapical puncture

**Figure 2:**
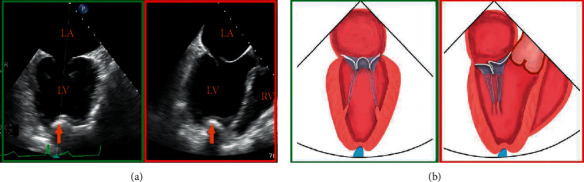
Determination of an appropriate transapical puncture site. (a) The notch of the fingertip (orange arrow) is displayed simultaneously on X-plane views and schematic diagram (b), and oriented toward the center of the valve coaptation.

To identify an appropriate puncture site, the interventional cardiologist can gently press the apex by fingertip. In detail, the notch of the fingertip is expected to be simultaneously displayed on the apical site of X-plane views and oriented toward the center of the MV orifice (Figure 2). The puncture site should afford a transapical route perpendicular to the annular plane.

**Figure 3:**
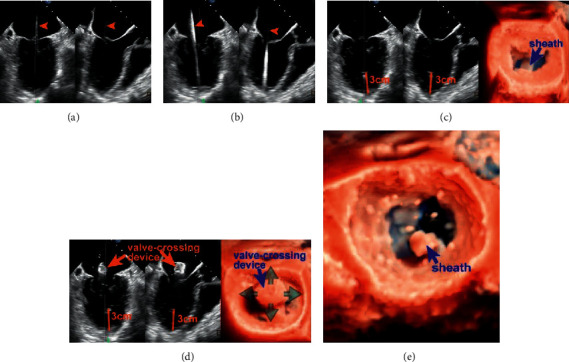
Transapical puncture and introducing the valve-crossing device. (a) X-plane view shows the guidewire enters LA through the mitral orifice (orange arrowhead). After that, a 6F guidewire is exchanged and advanced into LV and LA on a similar view. (b) Arrowhead. (c) 3D enface view + X-plane view show the introducer sheath is inserted coaxially to the LV and the insertion depth is approximately 3 cm (orange line). (d) The valve-crossing device is loaded into the introducer sheath and can be shifted in full directions (green arrows) within a reasonable scope of the MV orifice. (e) Coaxial advancement of the introducer sheath into the center of LA along the rod of valve-crossing device. AP: apex.

A double pledget-supported purse-string suture is made around the predetermined puncture site prior to introducing the puncture needle and guidewire into LV. Then, the guidewire is advanced into LV and LA at the diastolic phase under X-plane view and adjusted laterally/medially or anteriorly/posteriorly to the center of MV orifice. It will be easily identified on X-plane view and 3D enface LA MV view (Figure 3(a)).

A 6F guidewire is then exchanged and advanced into LV and LA. A similar view is used to visualize and adjust the guidewire to the center of the MV orifice, so as to further confirm the accuracy of the puncture site (Figure 3(b)).

The 16F introducer sheath is then inserted into LV via the 6F guidewire. Under X-plane view and 3D enface LA MV view guidance, the introducer sheath is slowly advanced into papillary muscle level. In general, the insertion depth is approximately 3 cm (Figure 3(c)).

**Step 3 .**

Introducing the valve-crossing device

The next step is to withdraw the 6F guidewire and load the valve-crossing device into the introducer sheath.

The valve-crossing device comprises a cylinder mesh made of a nitinol alloy and a stainless-steel rod. The cylinder mesh can produce elastic deformation when contacting with cardiac tissue, such as MV leaflet, chordae tendineae, and LA wall.

In order to exclude the interference of chordae tendineae, it is essential to identify possible deformation and resistance when passing the valve-crossing device through a subvalvular structure (Figure 3(d)).

If necessary, the interventionalist could withdraw the valve-crossing device back to the left ventricle and repeat the valve-crossing movement. This aims to further exclude the involvement of any chordae tendineae.

The following step is to advance the introducer sheath into the center of LA along the rod of the valve-crossing device while keeping the valve-crossing device steady. It is of critical importance to monitor the path of the introducer sheath inside the LA and the spatial relationship between the tip of the introducer sheath and LA wall using X-plane view or 3D enface LA MV view + X-plane view (Figure 3(e)). A recommended insertion depth is approximately 11–12 cm. After checking that the tip of the introducer sheath is in the center of LA, the valve-crossing device is retrieved.

**Step 4 .**

Advancement and adjustment of the clamp

The clamp is inserted into the introducer sheath and sent to the left atrium under X-plane view or 3D enface LA MV view + X-plane view monitoring. The clamp proceeds slowly until the opening of the proximal (front) clamp is visualized. Next, to open the rear clamp, the introducer sheath is carefully pulled back.

**Figure 4:**
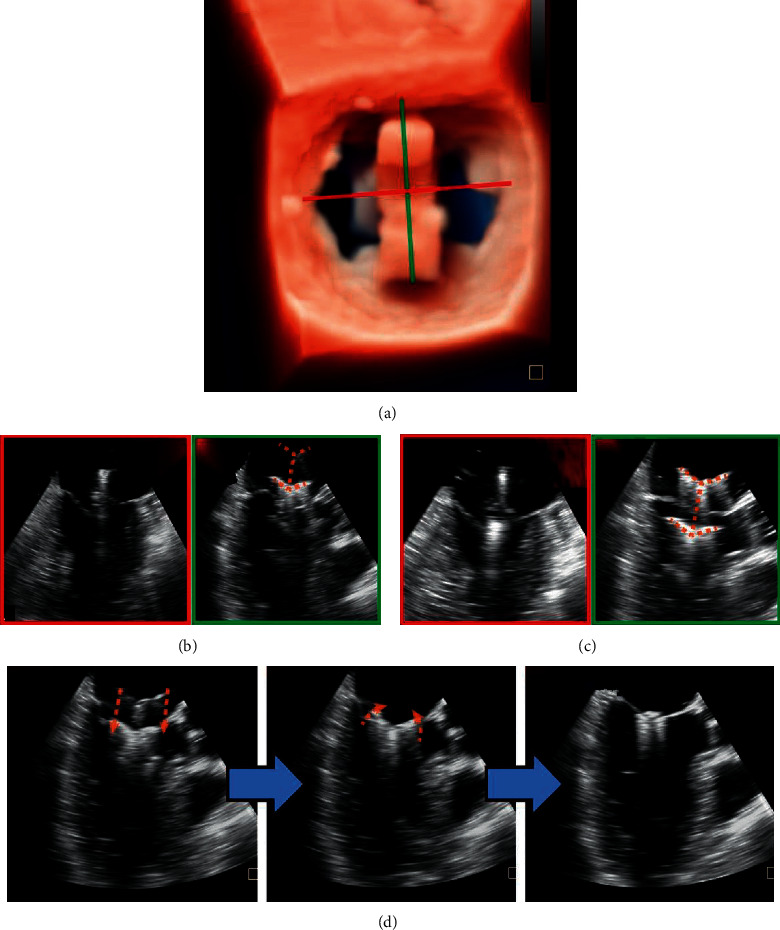
Advancement and adjustment of the clamp. 3D enface view (a) and X-plane view (b) show that the clamp is located in the center of the MV orifice and perpendicular to the MV closure line. The full length of the clamp arms (c) (orange dotted line) is seen in the long-axis view, and in the ME-commissural view, no clamp arms but the delivery rod should be seen. Next, the rear clamp is retracted into the LV, and the clamp orientation is reassessed with lowered gain settings (right: oblique perspective). (d) Leaflet Grasping.

The clamp then needs fine adjustment of (a) its position by shifting on the lateral-medial and anterior-posterior axis of the MV until it is above the target grasping area; (b) its perpendicularity to the MV orifice commissural line by rotating it clockwise or counterclockwise (Figure 4(b)); and (c) its splitting the MR jet in ME-commissural and LAX views. For central pathology, the full length of the clamp arms is seen in the midesophageal (ME) long-axis (LAX) view, and in the ME-commissural view, no clamp arms should be seen [17]. This position can be further confirmed using CFD to demonstrate the origin of the MR jet.

**Step 5 .**

Leaflet Grasping

After verification of its position and orientation, introducing by X-plane view or 3D enface LA MV view + X-plane view, the delivery system was withdrawn slowly so that the rear clamp was entering into the LV and the proximal clamp remained within the LA (Figures 1(e) and 4(c)). The orientation and position of the clamp have to be reassessed on X-plane views and 3D enface LA MV view, as the clamp may shift during translation from the LA to the LV. Once the clamp is in a satisfactory position, the delivery system was moved forward slightly so that the two targeted leaflets rest on each rear arm.

At this point, the proximal clamp is drawn back (Figure 4(d)) to capture the leaflets. The closing ring was moved forward and sleeved outside the clamp so that the clamping arms could approximate and close toward the central line.

ME-LAX view allows confirmation that (a) both leaflets are within the arms of the clamp and (b) the approximation of the proximal clamp has induced a full grasping of the leaflet.

The configuration of the clamp arms expands the range for leaflet grasp, which is considerably larger than that of MitraClip despite their arms' similar size. This characteristic may make it easier to grasp leaflets with ValveClamp and potentially affords a greater chance of procedural success especially in patients with a large flail gap. [18].

**Step 6 .**

Valve function assessment

**Figure 5:**
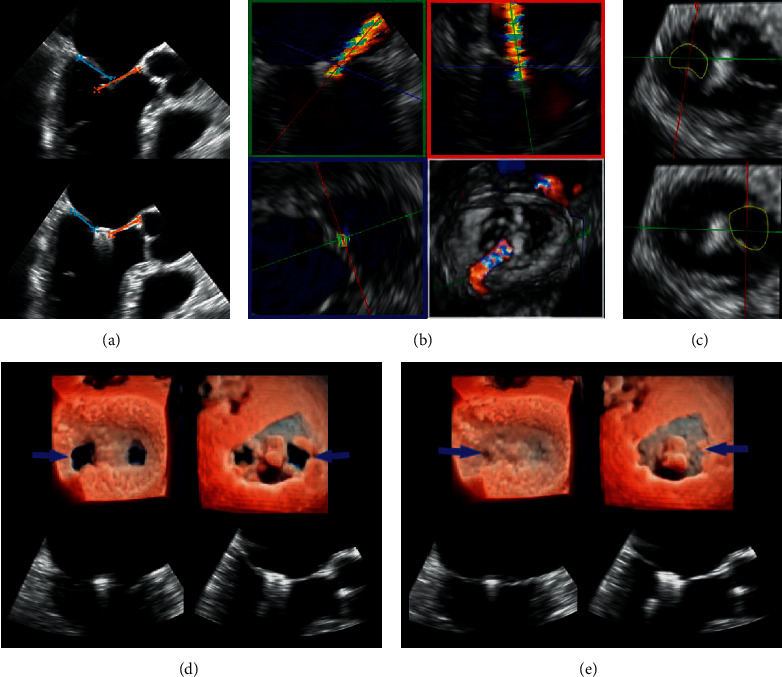
Valve function assessment. (a) The length of leaflet insertion can be calculated, which equals the difference between the length of leaflets before and after grasping. (b) Planimetry of vena contracta (VC) area in a wall-hugging residual regurgitant jet. (c) Using multiplanar reformatting, planimetry of each residual orifice will allow for the calculation of cumulative MVA to exclude mitral valve stenosis. Each orifice should be measured in separate planes as they are not in the same plane. (d, e) The newly created double orifices are assessed by 3D zoom MV enface LA and LV view + X-plane view in the systolic frame (d) and diastolic frame (e).

Assessment of the grasping effectiveness is of importance and depends entirely on 2D and 3D TEE. The assessment covers 4 aspects: length of leaflet insertion, severity of residual regurgitation, MV geometry, and degree of stenosis (Figure 5).

### 3.1. Length of Leaflet Insertion

Length of anterior and posterior leaflets of MV in the diastolic phase can be measured in the LAX view of X-plane view just before and after grasping, and the length of leaflet insertion can be obtained, which equals the difference between the length of leaflets before and after grasping. A minimum of 5 mm is deemed adequate for ValveClamp.

### 3.2. Residual Regurgitation

This assessment of residual regurgitation usually uses multimodal criteria per the current guideline, especially in case of incomplete correction of MR. [19] The presence of small color jets, even if multiple, is generally consistent with mild MR.

**Figure 6:**
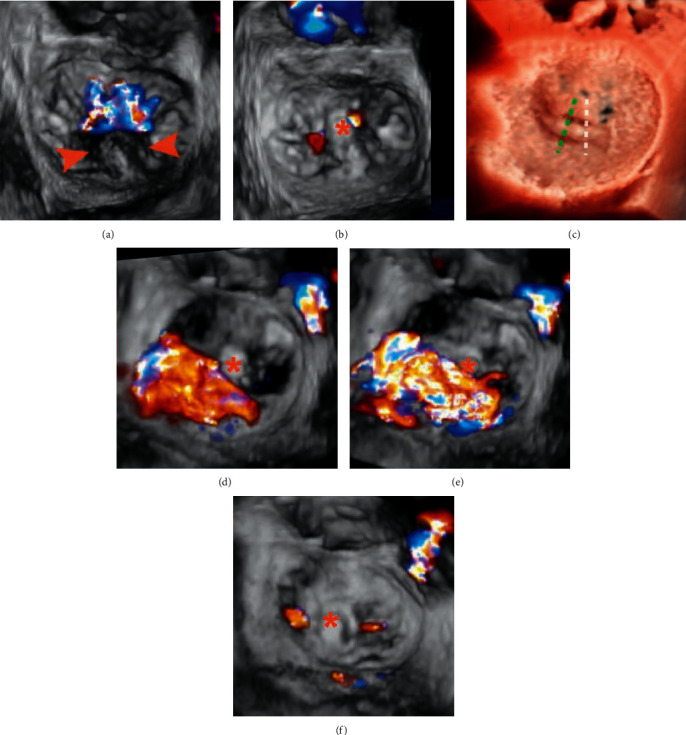
3D CFD TEE assessment Patient A: (a) 3D TEE image with CFD showing a prolapse P2 segment (arrowheads) with a single wide central jet and (b) after implantation of 1 clamp, trivial residual mitral regurgitation is visible. Patient B: (c), (e), (f) in a case of noncentral bileaflet prolapse, prerelease 3D CFD TEE (d): early systolic frame, E: mid systolic frame) revealed a significant lateral residual MR following a central implantation of 1 clamp (c: white dotted line); after adjustment of position and regrasping of leaflets (c: green dotted line), two trivial residual MR jets are visible (f). This case highlighted the importance of precise clamp deployment for the maximal reduction of MR. Asterisks indicate clamps.

In daily practice, 2D and 3D CFD imaging allow visual detection of residual mitral jets. The grading scheme of residual MR has yet to be validated, but the jet dimension into the left atrium is routinely screened to exclude significant regurgitation. 3D CFD helps the implanting team determine whether the clamp should be adjusted or additional clamp is indicated (Figure 6).

### 3.3. MV Geometry and Mitral Stenosis

In central MR jets, a symmetric double-orifice MV is typically created, and the residual mitral orifices areas can be measured through direct planimetry (Figure 5). The gradient across the MV is evaluated using CW doppler after (each) clamping to prevent significant mitral stenosis. Mean gradient ≤5 mmHg is acceptable. [20].

**Step 7 .**

Release of the clamp

If the above four criteria are satisfied, the clamp can be released. This process must be monitored using 2D and 3D TEE. At this point, the delivery rod that holds the clamp and valve must be steady to avoid leaflet tethering.

If the above four criteria are considered not satisfactory, the closed ring could be reversed to reopen the clamp. The rear clamp could be moved back to the LA during diastole and the above clamping steps then could be repeated.

## 4. Additional ValveClamp Implantation

**Figure 7:**
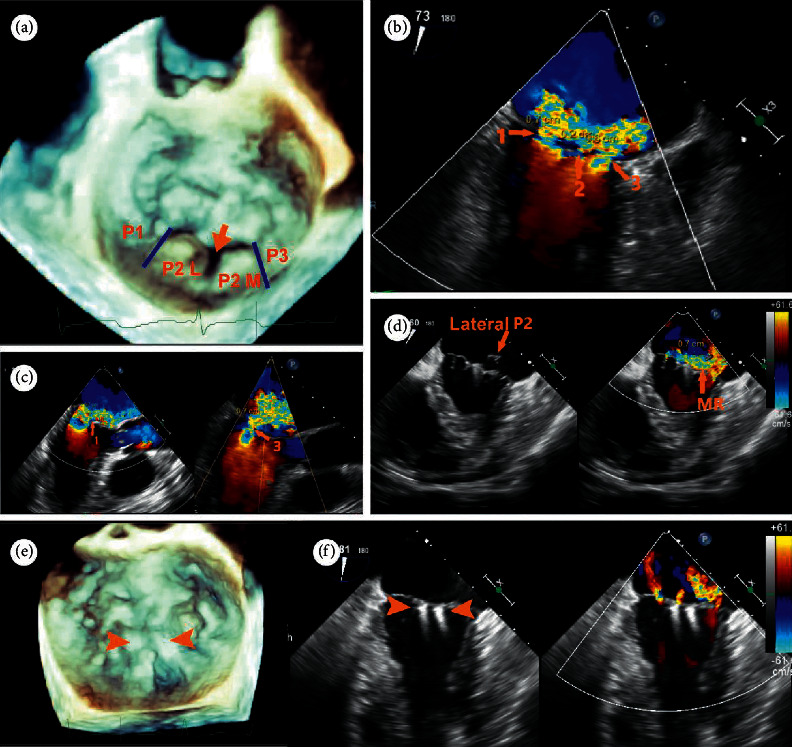
A 79-year-old male patient was referred for a history of chronic dyspnea (New York Heart Association functional class IV. (a) 3D enface view of the MV demonstrating a central cleft-like indentation with a prolapse lateral P2 (P2 (L) and a prolapse medial P2 (P2 (M). (b, c) 2D CDF TEE showing two dominant regurgitant jets (VCW: 7 mm and 6 mm, resp.) originating from the two prolapse P2 segments and a mild jet (VCW: 2 mm) from the central P2 indentation on bicommissural view and LVOT view. (d) Heart team decision was made on transapical ValveClamp implantation after his being deemed too high risk for surgical intervention. Implantation of the first clamp led to correction of the medial P2 segment, while the lateral P2 segment remained prolapse with a significant residual MR jet. (e) After implantation of the second clamp at the P2 segment, prolapse segments and regurgitant orifice were corrected and leaflet coaptation was preserved. (f) 2D CFD TEE showing mild residual jets (this case was diagnosed and treated before the TrueVue technique was commercially available).

If the residual MR is deemed moderate or more after deployment, additional clamps should be considered. The following criteria should be satisfied before the next implantation in our center: mitral valve opening orifice area ≥3.5 cm^2^, mean transmitral gradient ≤5 mmHg, and both anterior and posterior leaflet length>10 mm at the regurgitant site. Implantation of a second clamp should start with another valve-crossing manipulation.  Figure 7 presents a typical case of 2 clamps implantation.

## 5. Noncentral Mitral Regurgitation

**Figure 8:**
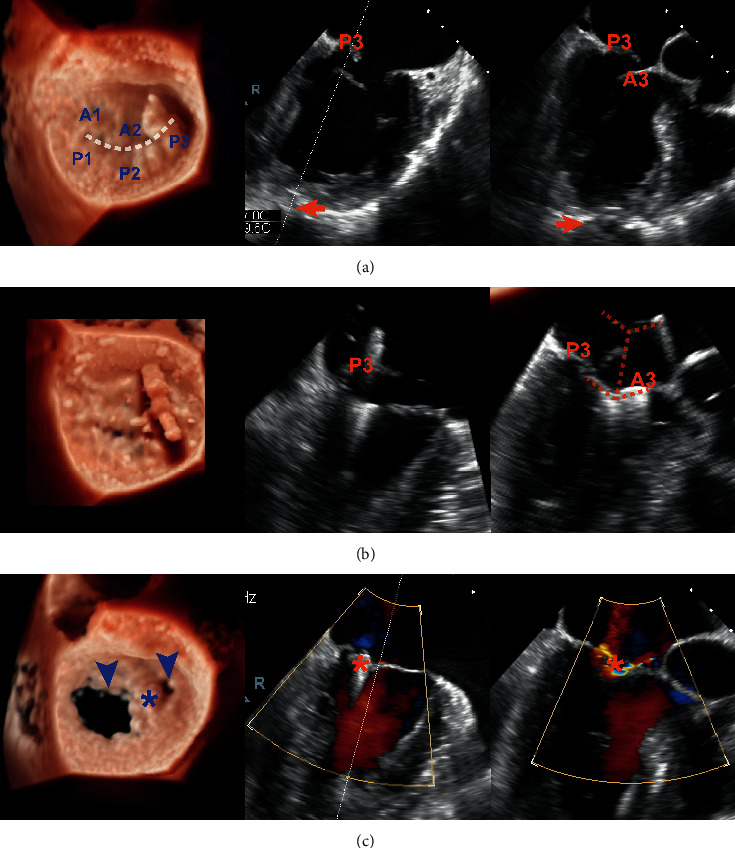
Noncentral ValveClamp implantation and its effect in a patient with a P3 prolapse and flail. Left column: real-time 3D TEE TrueVue mode; middle and right column: ME-commissural view and ME-LAX view of X-plane imaging mode, respectively. (a) X-plane image (systolic frame) helped the operator to identify a proper site of puncture (arrow). (b) The process of leaflet capture using the clamp (orange dotted line). (c) 3D TEE (diastolic frame) demonstrating a large and a small MV orifice (arrowheads) and X-plane image (systolic frame) showing two trivial residual regurgitant jets after clamp release (asterisks).

It is reported that approximately one-third of patients with dMR had noncentral dMR, highlighting the significant prevalence of noncentral MR [21, 22]. If MR originates from the lateral or medial part of the coaptation line (A1/P1 and A3/P3), the implantation process shares the identical principle with that of central MR; that is, the clamp should be aligned perpendicular to the line of coaptation and positioned ideally at the middle of regurgitant jets.  Figure 8 illustrates a typical case of noncentral implantation in a patient with prolapse and fail P3.

The X-plane angle of the initial ME-commissural view can be corrected by either subtracting 10–40° for A1/P1 pathologies or adding 10–40° for a prolapse in the A3/P3 segment. This will permit a perpendicular LAX view as the 90° angle between the biplane angles is maintained. [17].

The design and route of the ValveClamp system permit coaxial adjustment of perpendicularity and position of the clamp within the medial and lateral MV aspects. Care should be taken during this process to circumvent contact of the clamp arms with the LA wall. Also, leaflet length at the target site should be >10 mm.

## 6. Indications and Complications

Surgery represents the standard of care for dMR owing to excellent efficacy and long-term results of mitral valve repair. The current iteration of the ValveClamp system (via transapical access) was only used for patients with dMR with prohibitive surgical risk (generally due to age). A transapical E2E repair can be considered depending on the mitral valve anatomy evaluated by the interdisciplinary Heart Team.

The transapical access, compared to the percutaneous approach, may bring a higher degree of myocardial injury, especially in elderly patients with reduced LV ejection fraction preprocedurally, and harmful effects of thoracotomy according to the transcatheter aortic valve replacement literature [23, 24]. However, for ValveClamp, the delivery system (14–16F) via this approach was miniaturized to reduce the invasiveness of the procedure and decrease periprocedural complications. Moreover, a new generation using the transfemoral approach is under development and is expected to extend to patients with functional MR.

## 7. Conclusions

This review describes a systematic and easy-to-perform 2D and 3D TEE guidance protocol for transapical E2E repair. This protocol has several advantages compared to that of other transcatheter mitral valve repair devices: (1) this protocol entails only a few standard 2D and 3D views recommended by the current guideline, which would provide a steeper learning curve for echocardiographer and interventionalist and simplify the implantation steps; (2) it highlights the combination use of real-time 2D and 3D TEE imaging, instead of frequent probe manipulations and transpositions with 2D TEE, to consistently and precisely monitor and guide the procedure; (3) TrueVue real-time 3D imaging technique makes it simpler to visualize the spatial relationships between the delivery system and clamp, and the interventional objective and adjacent vital structures by providing images with tissue detail and depth perception, improving the confidence of image interpretation.
